# Possible Clinical Failure of Artemether-Lumefantrine in an Italian Traveler with Uncomplicated Falciparum Malaria.

**DOI:** 10.4084/MJHID.2011.041

**Published:** 2011-10-01

**Authors:** Ernestina C. Repetto, Antonio Traverso, Claudio G. Giacomazzi

**Affiliations:** 1 Infectious Diseases Unit, Regional Hospital U. Parini, Aosta, Italy; 2 Microbiology Laboratory, Regional Hospital U. Parini, Aosta, Italy

## Abstract

Artemisinin-combination therapies (ACTs) are recommended for the treatment of uncomplicated malaria in endemic areas with multidrug resistant *Plasmodium falciparum*. We report a case of possible artemether-lumefantrine clinical failure in an Italian traveler with uncomplicated *P. falciparum* malaria imported from Democratic Republic of Congo.

Many studies have reported artemisinin-combination therapies (ACTs) to be the best antimalarial drugs available, due to their efficacy and potential to lower the emergence of resistance.^1^,^2^ ACTs are recommended by the World Health Organization (WHO) for the treatment of uncomplicated malaria in endemic areas with multidrug resistant *Plasmodium falciparum*.^3^

A 32-year old Caucasian man, two days after coming back from one month travel business in Democratic Republic of Congo, was admitted to the Infectious Diseases Unit because of high fever, headache, nausea and epigastric pain started 48 hours before. He had not taken any prophylaxis for malaria. Upon admission patient's vital parameters were: body temperature 37,1°C, pulse rate 112/minute, blood pressure 120/60 mm Hg, peripheral oxygen saturation 99%. Blood test results are summarized in Table 1. Peripheral blood smear was positive for *Plasmodium falciparum* with 0,2% parasitemia. Chest X-Ray and electrocardiogram were normal. The combination drug Riamet® (arthemeter-lumefantrine 20/120 mg), bought in Switzerland, was started following manufacturer advice: 6 dose regimen of 4 tablets at 0, 6, 18, 30, 42, 54 hours after meals. Within 48 hours the patient was afebrile and blood smear was negative for Plasmodium. The patient was discharged after 72 hours in good clinical condition. After 14 days he was readmitted to the Infectious Diseases unit with high fever (39.6 °C) and headache, started 48 hours before. Laboratory tests showed mild leukocytosis and anemia: hepatic enzymes were substantially similar to his previous values.

Laboratory tests are shown in Table 1. Blood cultures were done using the BacT/ALERT system (bioMérieux®, Marcy l’Etoile, France) and blood smear was performed and resulted positive for *Plasmodium falciparum* with 2% parasitemia. The combination of oral quinine solfate 600 mg three times per day and doxycicline 100 mg twice per day was started and continued for 7 days. He was discharged after 48 hours and completed the combination treatment without adverse events. Blood cultures for bacteria and fungi were negative. A control of biochemical tests and blood smear done after 2 days showed resolution of anemia and negativity of parasitemia. The patient did not report other febrile episodes.

The association of arthemeter-lumefantrine is one of the most popular ACTs and is currently used in many countries: it is an effective blood schizonticidal drug, it is well tolerated and fast acting.^4^ Arthemeter rapidly reduces the parasite mass and resolves the symptoms while lumefantrine eliminates residual parasites. The efficacy of the combination mostly depends in the number of parasites remaining after arthemeter has been eliminated from the body and the duration of which lumefantrine plasma concentration exceeds the minimum inhibitor concentration against the parasites. No problems with absorption of arthemeter has been reported while co-administration with fatty food ensures maximum absorption of lumefantrine component^5^ and the wide variation in the pharmacokinetics of lumefantrine among individuals is known to influence the efficacy of artemether-lumefantrine combination.

We report a case of possible clinical failure of artemether-lumefantrine in an Italian traveler with imported uncomplicated *Plasmodium falciparum* malaria. To our knowledge one previous case of treatment failure of artemether-lumefantrine in *Plasmodium falciparum* malaria imported from Sierra Leone has been published so far.^6^ The authors hypothesized that this was due to low plasma concentration of lumefantrine component for its assumption without meals.

The combination drug artemether-lumefantrine is highly effective in Africa and published data indicated a good efficacy in Democratic Republic of Congo.^7^–^8^ Nonetheless falciparum recrudescence after ACTs were recently increasingly reported^8^–^10^ and high failure rates were seen in Cambodia (13,5% in 2006), where the emergence of lumefantrine resistance could not be excluded and could be explained by the cross resistance between mefloquine and lumefantrine.

According to WHO classification of treatment outcome in high-transmission areas (2009) our patient must be considered a late clinical failure. Our laboratory could not perform either an *in vitro* antimalarial susceptibility test or to measure blood antimalarial levels so the association therapy with oral quinine and doxycicline was chosen in the suspicion of inefficacy of the combination artemether-lumefantrine. Two hypothesis were made: the first was that the patient’s second episode was a re-infection rather than a recrudescence, being infected twice over 14 days before his return. As the association of artemether-lumefantrine is ineffective against exo-erytrocytic cycle, the first course treatment could have cured first malaria episode but could have been partially effective for the incubating *Plasmodium.* The second hypothesis was a recrudescence due to low plasma concentration of lumefantrine for an unrecognized patient’s defect in intestinal absorption rather than an incorrect drug assumption (in fact the patient properly took each dose after meal).

With increasing number of travelers into malaria endemic areas more attention should be paid in advising the patients to consult health practitioners if fever reappears after a full course of antimalarial treatment, to early diagnose recrudescences or reinfections.

## Figures and Tables

**Table 1.** Pre-treatment blood results.

	Admission 1	Admission 2
Leucocytes	4,8*10^3^/μ l	7,9*10^3^/μ l
Erythrocytes	5,39*10^6^/μ l	4,3*10^6^/μ l
Hemoglobin	16,9 g/dl	13,4 g/dl
Hematocrit	50,7%	38,9%
Platelets	130*10^3^/μ l	272*10^3^/μ l
AST	75 U/l	39 U/l
ALT	111 U/l	59 U/l
LDH	554 U/l	339 U/l
γ-GTP	227 U/l	200 U/l
Total bilirubin	2,29 mg/dl	1,45 mg/dl
Creatinine	0,95 mg/dl	0,72 mg/dl
C-reactive protein	13,7 mg/dl	2,3 mg/dl

AST: aspartate amino transferase; ALT: alanine amino transferase; LDH: lactic dehidrogenase; γ-GTP: gamma glutamil transpeptidase.
